# Time of diagnosis of congenital upper limb anomalies: a cohort study of 703 patients from a local registry in Norway

**DOI:** 10.2340/17453674.2026.46807

**Published:** 2026-09-17

**Authors:** Ida N SLETTEN, Mona I WINGE, Jarkko JOKIHAARA

**Affiliations:** 1 Division of Orthopaedic Surgery, Oslo University Hospital, Norway; 2 Department of Hand Surgery, Tampere University Hospital and Tampere University, Finland

## Abstract

**Background and purpose:**

Several European countries offer nationwide prenatal ultrasound screening for fetal anomalies. Few studies have examined prenatal detection of upper limb anomalies, and existing reports originate exclusively from high‑volume centers outside Europe. We aimed to assess the timing of diagnosis in patients with all Oberg–Manske–Tonkin diagnoses except tumorous dysplasias referred to the largest specialized unit in Norway, investigate prenatal detection rates, and explore associations between patient‑ and hospital‑related factors and prenatal detection.

**Methods:**

We extracted data on timing of diagnosis according to Oberg–Manske–Tonkin phenotype, and patient- and hospital-related variables from the CULA (congenital upper limb anomaly) North Oslo Registry from 2018 to 2025. For patients with anomalies detected prenatally or at birth, we analyzed associations between patient- and hospital-related factors and prenatal detection rate by multivariable logistic regression.

**Results:**

703 consecutive patients were enrolled. 402 (57%) patients had their anomaly detected at birth and 252 (36%) later in life. Prenatal ultrasonography detected the upper limb anomaly in 49 (7.0%) of the patients. Among 426 patients with anomalies visible at birth whose mothers had at least 1 prenatal ultrasound scan, the examination detected the anomaly in 49 (12%). Among phenotypes observed in more than 5 patients, congenital contractures (amyoplasia, distal arthrogryposis), reduction deficiencies (transverse, radial, ulnar), and ulnar polydactyly had the highest detection rates. Prenatally detected cases were more often born in university hospitals and more frequently had bilateral upper limb involvement and/or associated anomalies than those diagnosed at birth.

**Conclusion:**

7.0% of the upper limb anomalies were detected prenatally, 57% at birth, and 36% later in life. Prenatal detection rates were lower than those reported from larger non-European hand units, likely due to the inclusion of all upper limb anomaly diagnoses in this study and Norway’s decentralized prenatal care system.

Congenital upper limb anomalies (CULA) are rare [1]. They may be obvious at birth (e.g., polydactyly, syndactyly, severe limb reduction deficiencies) or become evident later in life (e.g., camptodactyly, Madelung deformity). Several CULAs can be detected at prenatal ultrasound [2-6]. The optimal window for visualizing the fetal hand and digits is the late first and early second trimesters, when the hands are sufficiently large, the digits are extended, and intrauterine space allows adequate imaging [2].

Two centers, in Australia and the USA, have reported prenatal CULA detection rates, ranging from 21% to 42% [4-6]. Higher detection rates have been associated with a positive family history, anomalies affecting the entire upper extremity, the presence of associated anomalies, and examinations performed in high-volume centers [3,4,6]. Norway, in contrast, is a sparsely populated country with few high-volume obstetric centers. No prior Norwegian data exists on prenatal CULA detection, and, to our knowledge, no registry‑based European studies have addressed this question.

The primary aim of our study was to investigate the timing of diagnosis in patients with CULA, and to assess prenatal detection rates for different CULA diagnoses. The secondary aim was to examine associations between patient‑ and hospital‑related factors and prenatal detection.

## Methods

### Study design

We included all CULA patients enrolled in the research component of the CULA North Oslo Registry with written consent from May 8, 2018, to May 7, 2025. We excluded patients with missing registry data concerning the time of CULA detection. For associations between prenatal CULA detection and patient- and hospital-related factors, we excluded patients with CULAs detected later in life and patients whose mothers did not have an ultrasound scan during pregnancy.

This study is reported according to the Strengthening the Reporting of Observational Studies in Epidemiology (STROBE) statement [7]. Our hand unit is a secondary referral center for CULA for the South-Eastern Norway Regional Health Authority, which serves a population of 3.1 million inhabitants, and is a tertiary referral center for the rest of the country. Except for a small number of minor cases, we treat all CULA patients in our geographical health region (55% of Norway’s population) and many patients with more severe CULAs from the other 3 Regional Health Authorities. In May 2018, we established the CULA North Oslo Registry [8,9] with a 2-fold quality and research design. At first attendance, all CULA patients are included in the registry and diagnosed by senior consultant congenital hand surgeons according to the Oberg–Manske–Tonkin (OMT) classification [10,11]. We include all diagnoses in the registry except for OMT IIIB phenotypes (dysplasias—tumorous conditions) [10]. The quality part of the registry includes information on CULA diagnoses, associated anomalies and syndromes, and treatments for all CULA patients assessed in our hand unit. Upon receipt of written consent for research from the caregivers or patients (if older than 16 years), we add additional background information concerning the family and pregnancy to the research component of the registry [8,9]. All background information (including a question regarding when the CULA was first detected) is collected with a structured questionnaire completed by the caregivers at the inclusion date. We delete all background data if we do not obtain re-consent from patients reaching the age of 16.

### Screening program

Nearly all pregnant women in Norway undergo at least 1 fetal ultrasound examination through the free, nationwide prenatal screening program. This program has, over the last 3 decades, consisted of a free-of-charge ultrasound examination during gestational weeks 17 to 19. In 2022, it was expanded to also offer an early screening during weeks 11 to 14 [12]. The pregnant woman is referred by her general practitioner or midwife to the nearest maternity ward or a maternity ward of choice. Despite a total population of only 5.5 million, Norway has 44 hospital-based obstetric units, reflecting long travel distances in rural areas, all of which are part of the public healthcare system [13]. Most ultrasound examinations are performed by a specially trained midwife at the nearest hospital where the birth is planned. Midwives who perform prenatal screening ultrasounds in Norway complete a formal, 1‑year postgraduate training program in obstetric ultrasonography [14]. This training combines structured theoretical instruction with extensive supervised clinical practice and includes systematic assessment of fetal anatomy across the first, second, and third trimesters. In most cases, the midwives interpret their own findings, and the report is stored in the mother’s medical record. Besides the national program, private clinics in urban areas also offer prenatal screening, performed by midwives or gynecologists. Regardless of the location of the primary examination, pregnant women with suspected fetal anomalies are referred to 1 of 5 prenatal diagnostic centers at regional university hospitals for further evaluation. At these centers, obstetricians with subspecialty expertise in fetal medicine perform high-resolution fetal ultrasonography and, in selected cases, additional prenatal diagnostic procedures, including non-invasive prenatal testing, chorionic villus sampling, amniocentesis, and fetal MRI [12].

### Variables

We extracted registry data concerning the patients’ Regional Health Authority and age at referral, caregiver-reported time of CULA detection, and assessor-reported OMT phenotypes for all included patients. Parents/caregivers reported CULA detection as follows: (i) detected on ultrasound, the prenatal diagnosis did not differ from the postnatal diagnosis; (ii) detected on ultrasound, the prenatal diagnosis differed from the postnatal diagnosis; (iii) detected at birth (missed on ultrasound); (iv) detected at birth (no prenatal ultrasound); or (v) diagnosed later (ranging from a few days to several years). Our CULA registry does not contain data on the number of ultrasound scans performed during the pregnancy, or on the examiner’s profession (midwife or physician). We do not have access to maternal medical journals, including those for patients born in our own hospital.

In patients with both OMT IA (proximal to the hand plate) and IB (hand plate) diagnoses describing the same phenotype, we designated the IA diagnosis as main (e.g., longitudinal deficiencies affecting the carpus/wrist/forearm as well as the hand, symbrachydactyly presenting with a shortened or narrowed forearm in addition to short, webbed fingers). For patients with bilateral CULA with different phenotypes (n = 29), we defined the main diagnosis as the phenotype that, despite optimal treatment, would have the largest impact on the patient’s ability to perform daily tasks.

### Outcome

For patients with a CULA visible at birth whose mothers had at least 1 prenatal ultrasound scan, we extracted caregiver-reported registry data on maternity hospital, plurality (singleton/multiple), positive family history of congenital anomalies (yes/no), and CULA (yes/no). We categorized the maternity hospitals into university or local/regional. As we did not have access to maternal medical records, we made the assumption that most pregnant women had at least 1 prenatal ultrasound in the same hospital where they gave birth, either because they lived within the hospital district or were referred due to pregnancy complications and/or findings of congenital anomalies at prenatal scanning in local hospitals. We extracted assessor-reported registry data on unilateral or bilateral CULA, along with associated anomalies and syndromes.

### Statistics

We presented categorical data as count numbers and proportions. Exact binomial 95% confidence intervals (Clopper–Pearson) for prenatal detection rates were used due to small sample sizes in several phenotypes. We reported continuous data as the median and interquartile range (IQR) due to the skewed data. We investigated associations between prenatal detection and patient- and hospital-related factors by multivariable logistic regression and presented both crude and adjusted odds ratios (ORs). Birth in a university hospital was included as the sole covariate in the adjusted analyses. It was selected a priori because it was the only available variable not intrinsically linked to the developmental or clinical pathway of CULA or associated anomalies and therefore represented an external factor that might influence the likelihood of prenatal detection.

### Ethics, data sharing plan, funding, use of AI, and disclosures

The Data Protection Officer at Oslo University Hospital approved the study (ID 25/15067), while the regional ethical committee waived approval (ID 910356). We conducted the study in accordance with the Declaration of Helsinki. The data underlying this study is not publicly available due to patient privacy restrictions. This research received no specific grant from any funding source. Grammarly and Microsoft Copilot were used to provide language support during the editing phase of the manuscript. The authors have no conflicts of interest to declare. Complete disclosure of interest forms according to ICMJE are available on the article page, doi: 10.2340/17453674.2026.46807

## Results

**Figure 1:**
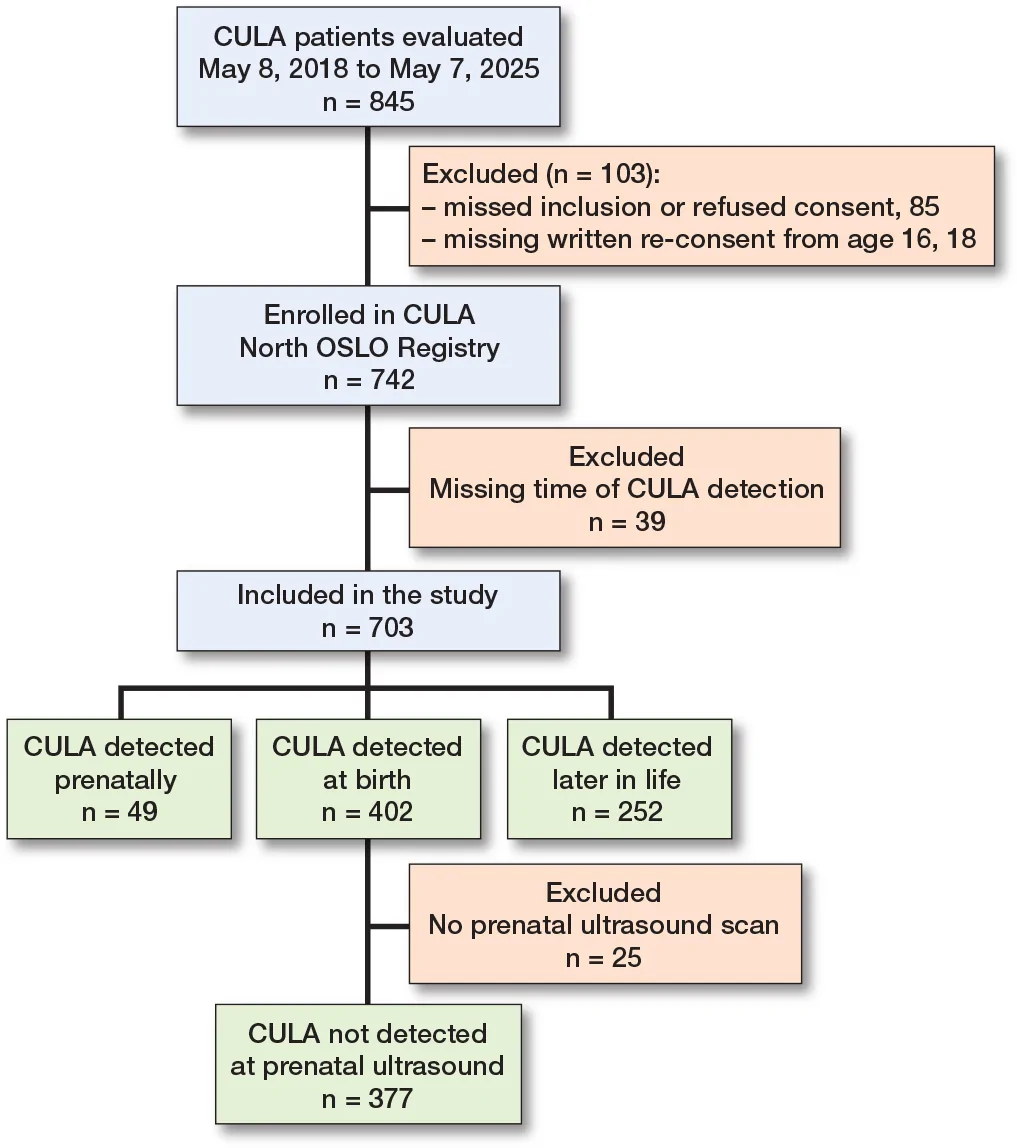
Flowchart.

During the study period, we included 845 consecutive patients in the quality-assurance component of the CULA North Oslo Registry (Figure 1). Caregivers provided consent and completed the background questionnaire for the research component of the registry for 760 (90%) patients. Of these, we included 703 (93%) in our analysis as we excluded 18 patients for whom re-consent at 16 years had not been obtained and 39 patients with missing data regarding time of diagnosis. In 49 (7.0%) patients, the CULA was detected prenatally, while it was detected at birth in 402 (57%), and later in life in 252 (36%). 615 referrals (87%) originated from the South-Eastern Regional Health Authority. The other 3 Regional Health Authorities contributed 8, 33, and 47 referrals, which did not reflect the regions’ population sizes. Most patients with a CULA detected prenatally or at birth were evaluated in our hospital within the first months of life. Patients with a CULA detected later in life generally had less severe anomalies (Table 1).

**Table 1:** Baseline characteristics (N = 703). Values are count or as indicated, median and interquartile range (IQR)

Factor	Detected prenatally (n = 49)	Detected at birth (n = 402)	Detected later (n = 252)
Age at diagnosis, median (IQR)	2 months (1–6.5)	3 months (1–13)	10 years (2.5–14)
Norwegian Regional Health Authority at referral			
South-Eastern	38	349	228
Central, Northern or Western	11	53	24
Distribution of Oberg–Manske–Tonkin phenotypes			
I Malformations			
IA Entire upper limb—Abnormal axis formation (early limb patterning)			
IA1 Proximal–distal axis			
IA1i Brachymelia with brachydactyly		1	1
IA1ii Symbrachydactyly spectrum (with ectodermal elements)	12	38	3
IA1iii Transverse deficiency (without ectodermal elements)		4	
IA2 Radial–ulnar (anterior–posterior) axis			
IA2i Radial longitudinal deficiency	8	37	37
IA2ii Ulnar longitudinal deficiency	2	12	3
IA2iii Ulnar dimelia		1	
IA2iv Radiohumeral synostosis		1	
IA2v Radioulnar synostosis	1	3	21
IA2vi Congenital dislocation of the radial head			6
IA2vii Forearm hemi-physeal dysplasia, radial (Madelung deformity), or ulnar		2	56
IA4 Unspecified axis			
IA4i Shoulder		1	2
IA4ia Undescended (Sprengel)		2	17
IA4ib Abnormal shoulder muscles			2
IB Hand plate—Abnormal axis differentiation (late limb patterning/differentiation)			
IB1 Proximal–distal axis			
IB1i Brachydactyly	1	25	36
IB1ii Symbrachydactyly (with ectodermal elements)	1	13	
IB1iv Cleft hand (split hand foot malformation)	1	12	
IB2 Radial–ulnar (anterior–posterior) axis			
IB2i Radial longitudinal deficiency, hypoplastic thumb			2
IB2ii Ulnar longitudinal deficiency, hypoplastic ulnar ray		4	
IB2iii Radial polydactyly		66	2
IB2iv Triphalangeal thumb			1
IB2iva Five-finger hand		1	
IB2vi Ulnar polydactyly	14	81	1
IB4 Unspecified axis			
IB4i Soft tissue			
IB4ia Cutaneous (simple) syndactyly		27	6
IB4ii Skeletal			
IB4iia Osseous (complex) syndactyly		7	
IB4iid Synostosis/symphalangism		1	2
IB4iii Complex			
IB4iiia Syndromic syndactyly (e.g., Apert hand)	1	2	
IB4iiib Synpolydactyly	1	8	
II Deformations			
IIA Constriction ring sequence	2	15	
IIB Not otherwise specified		1	
III Dysplasias			
IIIA Variant growth			
IIIA1 Diffuse (whole limb)			
IIIA1i Hemihypertrophy		1	2
IIIA2 Isolated			
IIIA2i Macrodactyly		4	1
IIIC Congenital contracture			
IIIC1 Amyoplasia multiplex congenital			
IIIC1i Amyoplasia	3	7	
IIIC1ii Distal arthrogryposis	2	8	2
IIIC2 Isolated			
IIIC2i Camptodactyly		11	46
IIIC2ii Thumb in palm deformity		6	3

The Oberg–Manske–Tonkin classification is simplified as this table includes only observed phenotypes in our study.

### Outcomes

**Figure 2:**
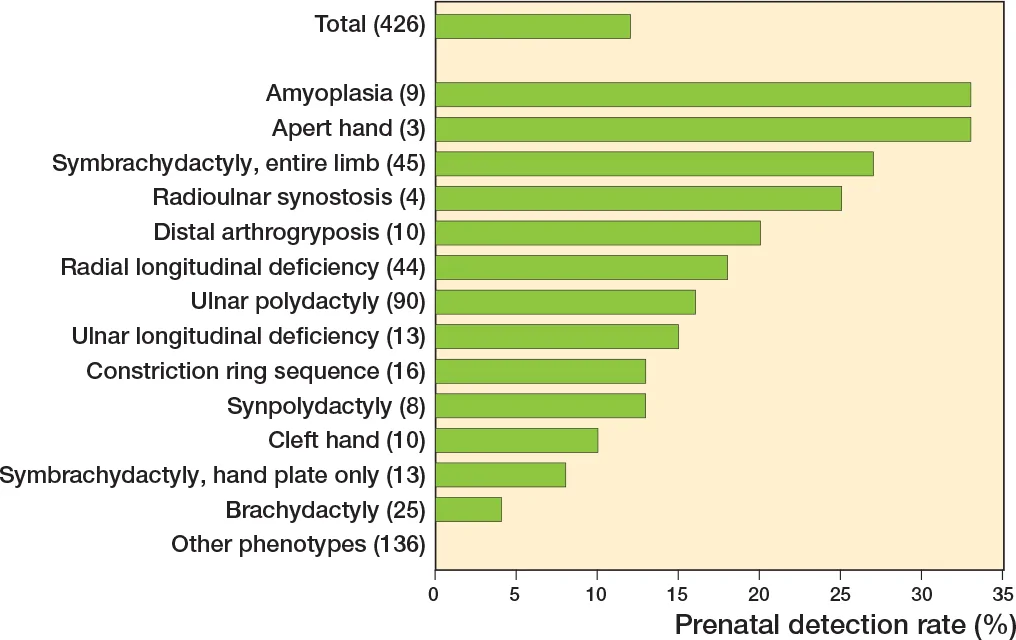
Congenital upper limb anomaly prenatal detection rates, overall and by phenotype. The total numbers of observed phenotypes are given in parentheses for each of the 13 phenotypes detected at prenatal ultrasound scans in the study population.

The overall prenatal detection rate of CULAs visible at birth was 49/426 (12%; Figure 2, Table 2). 13 OMT phenotypes were detected prenatally, with detection rates ranging from 4.0% to 33%. Among phenotypes with large enough sample sizes for meaningful interpretation, we found the highest detection rates for congenital contractures (amyoplasia and distal arthrogryposis), reduction deficiencies (transverse, radial, and ulnar), and ulnar polydactyly (Figure 2, Table 2). 12 of 13 patients with prenatally detected symbrachydactyly lacked the whole hand or at least 3 fingers. All 8 patients with prenatally detected radial longitudinal deficiency lacked the whole radius and/or the thumb. Of the 14 patients with ulnar polydactyly, 11 had a floating, pedunculated 6th finger, while 3 had a more fully formed extra digit. According to the caregivers, the prenatal CULA diagnosis did not differ from the postnatal diagnosis in 36 of 49 patients.

**Table 2:** Prenatal detection rates according to Oberg–Manske–Tonkin phenotype (n = 426)

		Prenatally detected, n	Total, n	Detection rate in % (CI)
All phenotypes		49	426	12 (8.6–15)
IA1ii	Symbrachydactyly, entire limb	12	45	27 (15–42)
IA2i	Radial longitudinal deficiency	8	44	18 (8.2–33)
IA2ii	Ulnar longitudinal deficiency	2	13	15 (1.9–45)
IA2v	Radioulnar synostosis	1	4	25 (0.6–81)
IB1i	Brachydactyly	1	25	4.0 (0.1–20)
IB1ii	Symbrachydactyly, hand plate only	1	13	7.7 (0.2–36)
IB1iv	Cleft hand	1	10	10 (0.3–45)
IB2vi	Ulnar polydactyly	14	90	16 (8.8–25)
IB4iiia	Apert hand	1	3	33 (0.8–91)
IB4iiib	Synpolydactyly	1	8	13 (0.3–53)
IIA	Constriction ring sequence	2	16	13 (1.6–38)
IIIC1i	Amyoplasia	3	9	33 (7.5–70)
IIIC1ii	Distal arthrogryposis	2	10	20 (2.5–56)

CI = 95% confidence interval.

NB: For all other Oberg–Manske–Tonkin diagnoses, the prenatal detection rate was zero.

Among the 426 patients with a CULA visible at birth, 402 were born in 35 different Norwegian obstetric units, and 24 were born abroad. Prenatal detection was associated with birth in a university hospital, bilateral CULA, and musculoskeletal as well as internal organ anomalies, and VACTERL (vertebral anomalies, anal atresia, cardiac defects, tracheoesophageal fistula, renal anomalies, limb anomalies) association, but not with a plurality or a positive family history of congenital anomalies (Table 3). Adjustment for maternity hospital produced little change in odds ratios, with wider confidence intervals due to missing birthplace data for 28 patients (Table 3).

**Table 3:** Family history and clinical findings in patients with a congenital upper limb anomaly (CULA) detected prenatally compared with patients with a CULA detected at birth (n = 426). Values are number of patients and proportions (%) unless otherwise indicated

Item	Detected prenatally (n = 49)	Detected at birth (n = 377)	Crude OR (CI)	Adjusted OR **^a^** (CI)
Caregiver-reported variables				
Birth in a university hospital **^b^**	33 (73)	167 (47)	3.1 (1.5–6.1)	
Twin or triplet **^c^**	5 (10)	18 (5)	2.3 (0.80–6.4)	2.2 (0.76–6.4)
Positive family history of				
congenital anomalies **^d^**	22 (46)	125 (34)	1.7 (0.92–3.1)	1.8 (0.93–3.3)
CULA **^d^**	14 (29)	88 (24)	1.3 (0.69–2.6)	1.3 (0.66–2.7)
Assessor-reported variables				
Bilateral CULA	30 (61)	169 (45)	1.9 (1.1–3.6)	2.1 (1.1–4.0)
Associated musculoskeletal anomalies	18 (37)	94 (25)	1.8 (0.94–3.3)	2.1 (1.1–4.0)
Lower limb	12 (24)	72 (19)	1.4 (0.68–2.8)	1.5 (0.76–3.2)
Spine	3 (6)	8 (2)	3.0 (0.77–11)	2.8 (0.69–11)
Associated other anomalies **^e^**	21 (43)	86 (23)	2.5 (1.4–4.7)	2.7 (1.4–5.1)
Heart	9 (18)	27 (7)	2.9 (1.3–6.6)	2.7 (1.2–6.3)
Syndrome	10 (20)	45 (12)	1.9 (0.88–4.1)	2.0 (0.90–4.3)
VACTERL association	4 (8.2)	7 (1.9)	4.7 (1.3–17)	4.5 (1.2–16)

a Adjusted for hospital type.

b Missing data (n = 28): born abroad (n = 21), out-of-hospital births (n = 7).

c Missing data (n = 1).

d Missing data (n = 5).

e All other anomalies besides the musculoskeletal system, including developmental delay.

CI = 95% confidence interval, OR = odds ratio, VACTERL = vertebral anomalies, anal atresia, cardiac defects, tracheoesophageal fistula, renal anomalies, limb anomalies.

## Discussion

Our study of 703 consecutive CULA patients provides new information on the time of diagnosis in a consecutive series of live-born children with CULA in a Western, sparsely populated country with a national prenatal screening program. We found that most patients had their CULA detected at birth, but a substantial proportion of patients were diagnosed much later in life. The overall prenatal detection rate was 12%, with the highest rates for congenital contractures, reduction deficiencies, and ulnar polydactyly. Patients with prenatally detected CULAs were more often born in a university hospital and presented more frequently with multiple congenital anomalies.

Not surprisingly, the prenatal CULA detection rate in our study population (12%) is lower than those reported from hand units in Australia and the USA for children with CULA. We assume this is mainly explained by the broad inclusion of all CULA phenotypes in our study in contrast to previous studies, including minor anomalies that are readily detected at birth but not during ultrasound scans, such as simple syndactyly and less severe reduction deficiencies (e.g., hypoplastic fingers including slight affection of forearm/wrist/carpus). An additional explanation might be the low population density and a decentralized healthcare system in Norway, in contrast to the high-volume, high-complexity hospitals where previous studies have been conducted. A registry-based Australian study reported an overall detection rate of 21%, but the registry included data on prenatal CULA detection for only 40% of the patients [5]. A study from the USA reported a detection rate of 31%, but it excluded minor CULAs that are difficult to detect on ultrasound [4]. The highest detection rate (42%) was reported in a study from the USA at a highly specialized tertiary referral center, where obstetrics and gynecology consultants performed all ultrasound examinations, and maternal-fetal medical specialists interpreted them [6]. It cannot be expected that less experienced assessors working in small obstetric units in rural areas in Norway obtain similar detection rates. However, the actual detection rate of CULA in the Norwegian national screening program is probably higher than 12%. Termination of pregnancies due to fetal anomaly (TOPFA) is not uncommon in Norway when multiple fetal anomalies are detected and has also been reported in cases of isolated limb reduction defects [15].

In our study, 73% of the parents confirmed the accuracy of the prenatal CULA diagnosis, which is comparable to the 58% reported in a USA study [4]. Similar to our findings, previous studies have indicated that amyoplasia, reduction deficiencies, and polydactyly have the highest prenatal detection rates [4-6], and that ulnar polydactyly is more often detected than radial polydactyly [4,5]. Despite this, it surprised us that ultrasounds failed to detect all 66 radial polydactylies and most reduction deformities.

Our finding that more CULAs were detected prenatally among patients born in university hospitals aligns with previous studies [3,6]. We could not assess associations between detection rates and Regional Health Authorities, as the number of referrals from the three Regional Health Authorities besides the South-Eastern reflected the local CULA surgical expertise more than the region’s population. Twin and triplet pregnancies involve more frequent ultrasound examinations, yet in our study, they were not associated with a higher prenatal CULA detection rate. This may reflect the small number of non-singletons in our study population, but it could also indicate the greater difficulty of visualizing all limb structures in multiple pregnancies. We did not find a higher prenatal detection rate in patients with a positive family history of CULA, despite its high prevalence in the study population. This may reflect that a large proportion of inherited CULA phenotypes visible at birth are minor finger defects that are not easily detected on prenatal ultrasound [16].

We found a higher prenatal detection rate in patients with bilateral CULAs than in those with unilateral CULAs. Many severe unilateral reduction deficiencies were not detected, possibly because the normal hand was described twice. Our study confirms previous findings of a higher detection rate in patients with associated anomalies, which, in turn, leads to a more meticulous upper limb examination [3,4,6,17]. Both findings are consistent with previous reports of a doubled likelihood of bilateral radial longitudinal deficiencies when part of a syndrome [17], and higher perinatal mortality in patients with bilateral limb anomalies [18,19]. VACTERL association had the strongest association with prenatal detection, which was not surprising as these patients often have multiple major anomalies of the internal organs.

In our study, only a small proportion of parents became aware of the CULA before birth, even for proximal deficiencies. A recent UK survey demonstrated considerable variation in parental preferences regarding whether a CULA diagnosis should be made prenatally or postnatally [20]. Regardless of timing, parents often fear associated undiagnosed anomalies, blame themselves, and worry about their child’s future physical and social functioning [20]. These concerns underscore the importance of congenital upper limb services in providing clear information regarding known associated anomalies, underlying conditions, and the generally high psychosocial functioning seen in children with CULA [21,22]. Our findings on time of diagnosis and prenatal detection rates provide clinicians with important, evidence-based expectations to communicate to families.

### Strengths

The strengths of our study included comprehensive and structured data collection and CULA classification by senior consultant congenital hand surgeons over 7 years, with few missing data. The coverage of the research registry was high, and selection bias was minimal, as the registry included patients with all CULA diagnoses except tumorous dysplasias, ensuring high internal validity of the study. The external validity (for areas with similar demographics and health systems) was also high, as 87% of the patients in this study were consecutive admissions from a defined geographical region.

### Limitations

The main limitation of our study was the unavailability of retrieving ultrasound reports from maternal medical records for verification of the caregiver-reported information concerning prenatal CULA diagnoses. Therefore, our study may suffer from parental misinterpretation bias or parental recall bias. Considering the median patient age of only a few months at registry enrollment, we assumed that caregiver‑reported information on whether a CULA was diagnosed at an ultrasound scan or not was sufficiently reliable for this study’s purpose, given the emotional impact typically associated with a prenatal anomaly diagnosis. On the other hand, parental assessments of the accuracy of the prenatal diagnosis may be less reliable. Obtaining ultrasonographic reports would have required additional written consent at the time of registry enrollment, and this procedure was not incorporated into the registry’s initial design. We considered retrospective collection of this data infeasible, as requiring new written consent from all 426 mothers would likely have caused response bias compromising the study’s sample size. Another study limitation was the relatively small study population, reflecting a sparsely populated country. Several rare phenotypes’ detection ranges had wide confidence intervals due to small sample sizes and must be interpreted with caution. Estimates for overall detection rate and for common phenotypes were considered acceptably precise.

### Conclusion

7.0% of the upper limb anomalies were detected prenatally, 57% at birth, and 36% later in life. Prenatal detection rates were lower than those reported from larger non-European hand units, likely due to the inclusion of all upper limb anomaly diagnoses in this study and Norway’s decentralized prenatal care system.

## Notes

All authors designed the study protocol. MIW and INS collected data. INS analyzed data, and all authors interpreted the findings. INS wrote the first draft of the manuscript, and all authors participated in manuscript editing.

The authors would like to thank research coordinators Elise Berg Vesterhus and Silje Stuksrud from the Division of Orthopaedic Surgery at Oslo University Hospital for their administrative assistance.

Handling co-editors: Ilkka Helenius and Robin Christensen.

*Acta* thanks Eeva Astrid Koskimies-Virta and other anonymous reviewers for help with peer review of this manuscript.
